# Emerging role of TRP channels in cell migration: from tumor vascularization to metastasis

**DOI:** 10.3389/fphys.2013.00311

**Published:** 2013-11-05

**Authors:** Alessandra Fiorio Pla, Dimitra Gkika

**Affiliations:** ^1^Department of Life Sciences and Systems Biology, Nanostructured Interfaces and Surfaces Centre of Excellence, University of TorinoTorino, Italy; ^2^Inserm U1003, Equipe labellisée par la Ligue Nationale contre le cancer, Université des Sciences et Technologies de LilleVilleneuve d’Ascq, France

**Keywords:** cancer cells, tumor angiogenesis, cell migration, TRP channels, Orai/Stim1

## Abstract

Transient Receptor Potential (TRP) channels modulate intracellular Ca^2+^ concentrations, controlling critical cytosolic and nuclear events that are involved in the initiation and progression of cancer. It is not, therefore, surprising that the expression of some TRP channels is altered during tumor growth and metastasis. Cell migration of both epithelial and endothelial cells is an essential step of the so-called metastatic cascade that leads to the spread of the disease within the body. It is in fact required for both tumor vascularization as well as for tumor cell invasion into adjacent tissues and intravasation into blood/lymphatic vessels. Studies from the last 15 years have unequivocally shown that the ion channles and the transport proteins also play important roles in cell migration. On the other hand, recent literature underlies a critical role for TRP channels in the migration process both in cancer cells as well as in tumor vascularization. This will be the main focus of our review. We will provide an overview of recent advances in this field describing TRP channels contribution to the vascular and cancer cell migration process, and we will systematically discuss relevant molecular mechanism involved.

## Introduction

Metastasis is the main cause of mortality in cancer and depends on two key processes: cell migration of cancer cell to invade adjacent tissues followed by intravasation into blood/lymphatic vessels and tumor vascularization, which give access to bloodstream. During the metastatic process cell migration of both epithelial and endothelial cells (EC) is an essential step leading to the spread of the primary tumor and to the invasion of neighboring connective tissue, lymphatic system and blood vessels. Cell migration and tumor vascularization are often accompanied by changes in ion channel expression and/or activity and, consequently, by abnormal progression of the cellular responses with which they are involved. In particular Ca^2+^ channels are of utmost importance since Ca^2+^ is the key messenger regulating signaling pathways important in cellular processes as proliferation, apoptosis, gene transcription, migration and angiogenesis (Roderick and Cook, 2008; Monteith et al., 2012).

In this context the relatively recent Ca^2+^ channel family of Transient Receptor Potential (TRP) have been associated with several cancers and their role has been increasingly clarified the two last decades (Bödding, 2007; Nilius et al., 2007; Gkika and Prevarskaya, 2009; Prevarskaya et al., 2011). TRP proteins display an extraordinary diversity of functional properties and have profound effects on a variety of physiological and pathological conditions (Ramsey et al., 2006; Nilius et al., 2007; Montell, 2011). Indeed TRP channels modulate intracellular Ca^2+^ concentrations, controlling critical cytosolic and nuclear events that are involved in the initiation and progression of cancer. It is not therefore surprising that the expression and function of some TRP channels is altered during tumor growth and metastasis. In particular, a typical feature of TRP channels is their ability to be activated by a wide range of external stimuli (including light, sound, chemicals, temperature, and touch) as well as of changes in the local environment (Venkatachalam and Montell, 2007; Nilius and Owsianik, 2011). As such, they can be envisioned as polymodal molecular sensors suggesting that the physiologically relevant stimulus for any given TRP will be governed by the specific cellular context (i.e., phosphorylation status, lipid environment, interacting proteins and concentration of relevant ligands), which dramatically changes during carcinogenesis. Indeed, recent evidences increasingly clarify the role for different TRP channels making them very promising players since their expression and/or activity mark and regulate specific stages of cancer progression (Nilius et al., 2007; Gkika and Prevarskaya, 2011; Prevarskaya et al., 2011; Ouadid-Ahidouch et al., 2013).

On the other hand, TRP channels are widely expressed in ECs and their functions have been related to critical steps of tumor vascularization as well as *in vivo* angiogenesis (Fiorio Pla et al., 2012a; Munaron et al., 2013). TRP channels-mediated Ca^2+^ influx can be triggered by the release from intracellular Ca^2+^ stores giving rise to store-operated Ca^2+^ entry (SOCE). An alternative route is second messenger, store-independent Ca^2+^ entry (NSOCE) (Ambudkar and Ong, 2007).

Due to the essential role of cell migration of both epithelial and EC in the so-called metastatic cascade that leads to the spread of the disease within the body, we provide here an overview of recent advances in this field describing TRP channels contribution to migration process systematically discussing relevant molecular mechanism involved.

## TRPC channels

TRPC channels are tetrameric, non-selective cation channels, which are central constituent of both store-operated Ca^2+^ entry (SOCE) as well as receptor-activated Ca^2+^ entry (ROCE). TRPC channels have been described to be functionally coupled to different tyrosine kinase receptor (i.e., VEGF, bFGF) and G protein-coupled receptors (Ambudkar and Ong, 2007).

Increasing evidences show the involvement of these channels in chemotaxis and directional migration processes (Schwab et al., 2012).

### TRPC1

The role of TRPC1 in cell migration has been shown by several groups. In particular TRPC1 channels determine polarity and persistence of different cell types and are involved in stimuli-mediated directional cues in both *in vivo* and *in vitro* (Wang and Poo, 2005; Fabian et al., 2008; Schwab et al., 2012).

**Figure 1:**
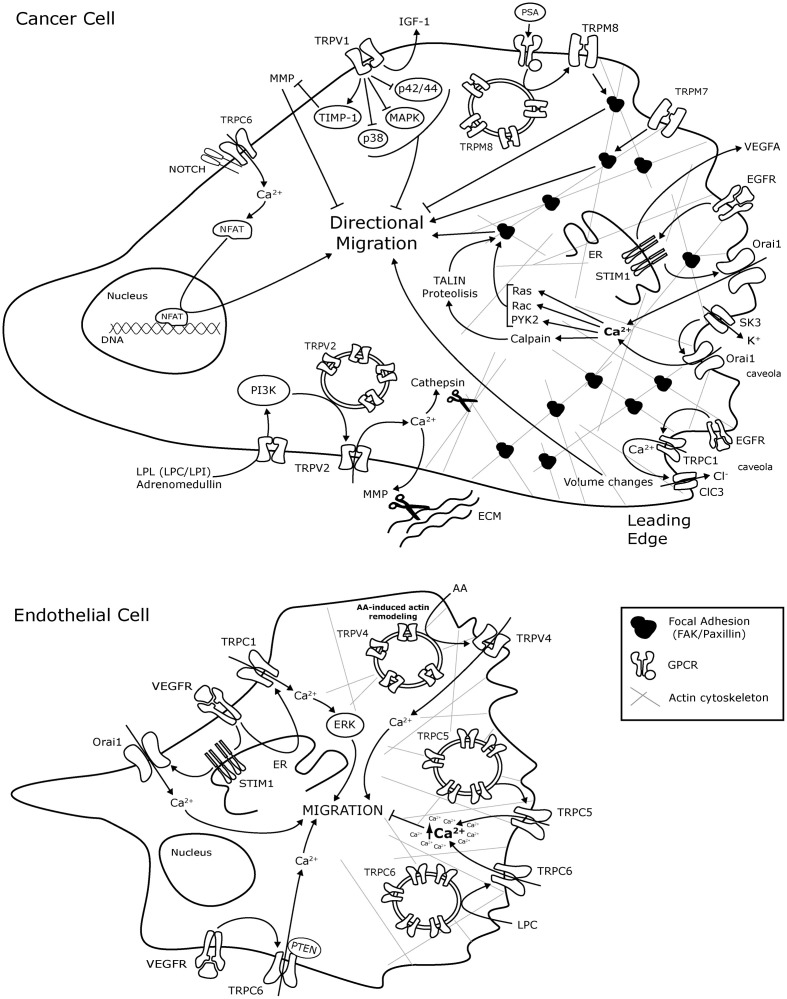
**Schematic representation of TRP and ORAI1 channels molecular mechanisms involved in cancer cell and endothelial cell migration.** The mechanisms are presented in representative Cancer cells and endothelial cells without any tissue specification. AA, arachidonic acid; ClC-3, Chloride channel-3; EC, endothelial cells; ER, endoplasmic reticulum; bFGF, basic Fibriblast Growth Factor; GZMA, Granzyme A; GPCR, G-protein coupled receptor; IGF, insulin-like growth factor; LPL, lysophospholipids; LPC, lysophosphatidylcholine; LPI, lysophosphatidylinositol; MAPK, mitogen activated protein kinase; MMP, Matrix metalloproteinase; MMP2, Matrix metalloproteinase 2; NFAT, Nuclear factor of activated T-cells; PI3K, Phosphatidylinositol 3-kinase; PTEN, Phosphatase and tensin homolog; Pyk2, Protein tyrosine kinase Pyk2; SK3, K + channel; TIMP1, metallopeptidase inhibitor 1; VEGF, Vascular Endothelium Growth Factor; VEGFR, VEGF Receptor.

As concerning cancer cell migration, TRPC1 is expressed in several glioma cell lines, including D54, D65, GBM62, STTG1, U87, and U251 and in Grade IV malignant glioma patient tissue (Bomben and Sontheimer, 2008). In glioma cells TRPC1 has been correlated with EGF-mediated directional migration. In particular EGF-mediated chemotactic migration is lost when TRPC channels are inhibited pharmacologically and reduced when the expression of TRPC1 is compromised through shRNA knockdown. Interestingly, TRPC1 channels localize to the leading edge of migrating glioma cells where they co-localize with markers of caveolar lipid rafts. This raft association appears important since disruption of lipid rafts by depletion of cholesterol impaired TRPC1channel-mediated Ca^2+^ entry and EGF mediated chemotaxis (Bomben et al., 2011) (Table 1). Interestingly TRPC1-mediated Ca^2+^ entry seems to colocalize with Chloride Channel ClC-3 in caveolar lipid rafts of glioma cells. This interaction is functionally relevant during EGF-induced chemotaxis. Therefore the authors propose that Cl^−^ channels (most likely ClC-3) are important downstream target of TRPC1 in glioma cells, coupling elevations in [Ca^2+^]i to the shape and volume changes associated with migrating cells (Cuddapah et al., 2013) (Table 1; Figure 1).

**Table 1:** **TRP/Orai1 functions in cancer and endothelial cell migration**.

**Channel**	**Cell type**	**Cell migration**		**Techniques used**	**Proposed mechanism**	**References**
		**Epithelial**	**Endothelial**			
TRPC1	Glioma cell lines; zebrafish; HUVEC	+	+	Boyden chamber; morfolinos on zebrafish; tubulogenesis *in vitro*	EGF-mediated migration, involvement of lipid raft, ClC-3 interaction/ filopodia extention	Yu et al., 2010; Bomben et al., 2011; Antigny et al., 2012; Cuddapah et al., 2013
TRPC3	MCF-7 (breast cancer)	+	ND	Boyden chamber	Indirect link	Zhang et al., 2012
TRPC6	Head and neck carcinoma cell line; glioblastoma multiple; HMEC; HPAEC; HUVEC;	+	+	Wound healing; matrigel invasion assays on transwell; soft agar colonogenic assay; tubulogenesis in vitro	Notch activation under hypoxia in turn promote TRPC6 expression; in EC PTEN regulates TRPC6 expression	Hamdollah Zadeh et al., 2008; Ge et al., 2009; Chigurupati et al., 2010; Kini et al., 2010; Bernaldo de Quirós et al., 2013
TRPC5/ TRPC6	BAEC, MAEC	ND	−	Wound healing	Lysophosphatidylcholine activate TRPC6 which in turn promote TRPC5 membrane expression	Chaudhuri et al., 2008
TRPV1	Human hepatoblastoma cells (HepG2); cervical and bladder cancer cell	±	−	Random cell migration, boyden chamber, matrigel invasion assays, in vivo xenografts on nude mice	TRPV1 antagonist capsazepine inhibits both cannabidiol-induced tissue inhibitors of the matrix metalloproteinase 1 (TIMP-1) expression and activation of the MAPKs p38 and p42/44; capsaicin promotes IGF (insulin-like growth factor)-1 release, GZMA and MMP9 activation, a-tubulin disassembly and cytoskeleton degradation. The effect is reverted by TRPV1 overexpression	Waning et al., 2007; Ramer and Hinz, 2008; Ramer et al., 2010; Caprodossi et al., 2011
TRPV2	Prostate cancer cells (PC3), urothelial cancer vells (T24/83)	+	ND	Migration assays on transwell, matrigel invasion assays on transwell, in vivo xenografts on nude mice	Lysophospholipids and adrenomedullin activate TRPV2 via PI3K pathway. TRPV2 activation induce MMP2, MMP9 and cathepsin B52 expression	Monet et al., 2010; Oulidi et al., 2013
TRPV4	Hepatoblastoma cell line (HepG2); bovine capillary endothelial (BCE) cells and human dermal microvascular endothelial (HMVE); BHMEC; TEC	+	+	Random motility; wound healing; live cell microscopy after mechanical shear stress application	Ultrarapid activation by b1integrin; activation by AA via actin remodeling	Thodeti et al., 2009; Matthews et al., 2010; Fiorio Pla et al., 2012b
TRPM7	Breast cancer, lung cancer, nasopharingeal cancer and pancreatic ductal adreno-	+	ND	Matrigel invasion assays on transwell	TRPM7 activation of Src-MAPK signaling pathway, focal adhesion number; EGF-mediated TRPM7 membrane expression	Gao et al., 2011; Middelbeek et al., 2012; Meng et al., 2013
TRPM8	Carcinoma prostate metastatic cancer; glioblastoma; squamous carcinoma cell lines	−	−	Wound healing; transwell; random motility; *in vivo* xenografts on nude mice	Activation by icilin and PSA; TRPM8 diminish PFAK levels	Wondergem et al., 2008; Yang et al., 2009b; Gkika et al., 2010; Zhu et al., 2011; Okamoto et al., 2012; Valero et al., 2012
ORAI1/ STIM1	Breast cancer; cervical cancer; HUVEC; EA.hy926 cells; EPC	+	+	Transwell; matrigel invasion assays on transwell random migration; *in vivo* xenografts on nude mice; *in vitro* tubulogenesis; wound healing	Stimulation of focal adhesion turnover via ras and rac GTPases; downstream to VEGF.	Abdullaev et al., 2008; Yang et al., 2009a; Chen et al., 2011; Dragoni et al., 2011; Li et al., 2011; Beech, 2012

HMEC, human microvascular EC; HPAEC, human pulmonary artery EC; HUVEC, human umbilical vein EC; EA.hy926, EC line derived from HUVECs fused with human lung adenocarcinoma cell line A549; BTEC, tumor derived EC from breast carcinoma; MAEC, Mause Aortic EC; BHMEC, brain microvascular EC; EPC, endothelial precursors cells; RCC-EPC, EPC isolated from renal carcinoma patients; EGF, epithelial Growth Factor; ClC-3, chloride channel; PTEN, phosphatase and tensin homolog protein; TIMP1, metallopeptidase inhibitor 1; MAPK, mitogen activated protein kinase; IGF, insulin-like growth factor; GZMA, Granzyme A; MMP9, Matrix metalloproteinase 9; PI3K, Phosphatidylinositol 3-kinase; MMP2, Matrix metalloproteinase 2; AA, arachidonic acid.

Beside the described role on cancer cell migration, a proangiogenic role for TRPC1 has been described *in vivo*. Knockdown of zebrafish TRPC1 by morpholinos causes severe angiogenic defects in intersegmental vessel sprouting, presumably due to impaired filopodia extension and EC migration. Furthermore, *in vivo* time-lapse imaging of cellular behaviors showed that the angiogenic defect caused by TRPC1 deficiency is associated with markedly impaired filopodia extension, migration, and proliferation of intersegmental vessels (ISV) tip cells (Yu et al., 2010) (Table 1). On the other hand TRPC1 is expressed in different endothelial cell types, and promotes capillary-like tube formation in primary human umbilical vein EC (HUVEC) cells but not on EA.hy926 cells, an endothelial cell line derived from HUVECs fused with human lung adenocarcinoma cell line A549 (Antigny et al., 2012) (Table 1).

Beside the role of resident (EC), great interest has been recently focused on circulating endothelial progenitor cells (EPCs), a subpopulation of bone marrow-derived mononuclear cells also found in peripheral blood as important players in tumor vascularization. They promote vessel formation in adult, and it was recently reported that they have the ability to incorporate into tumor tissues (Carmeliet, 2005; Bussolati et al., 2011). In this regard, TRPC1 has been suggested to contribute to regulation of cell migration of EPCs isolated from rats bone marrow. Interestingly TRPC1 downregulation dramatically reduces SOCE in EPC (Kuang et al., 2012) (Table 1). These data are in accordance with data obtained from EPC isolated from peripheral blood of patients affected by renal cellular carcinoma (RCC; RCC-EPCs) and control EPCs (N-EPCs). In this study in fact Lodola and co workers report that TRPC1 is upregulated in RCC-EPCs where it’s involved in SOCE (Lodola et al., 2012). On the other hand no data are at the moment available for a direct role for TRPC1 in human EPC.

In conclusion, form the data available, TRPC1 seems to be a general player involved in both cancer as well as endothelial cell migration although the molecular mechanism is still elusive.

### TRPC3

Not many data are available about the role of TRPC3 in cancer cell or EC migration. However recently a functional expression of TRPC3 has been described in MCF-7 breast cancer cell line. TRPC3 mediates store-operated Ca^2+^ entry (SOCE) as shown by TRPC3 knock down. Moreover PUFA (both arachidonic acid (AA) and Lysophosphatidic Acid) inhibits TRPC3-mediated Ca^2+^ entry, which correlates with inhibition of MCF-7 migration. The data suggest a possible role for TRPC3-mediated Ca^2+^ entry in breast cancer cell migration although a direct link is still missing (Zhang et al., 2012) (Table 1).

### TRPC5—TRPC6

TRPC6 was known till recently to increase proliferation of epithelial cells in prostate (Thebault et al., 2006; Yue et al., 2009), breast (Guilbert et al., 2008; Aydar et al., 2009), liver (El Boustany et al., 2008) and renal cancer (Song et al., 2013). Since TRPC6 protein over expression in breast cancer is not correlated with tumor grade, estrogen receptor expression or lymph node positive tumors (Guilbert et al., 2008), one could think that TRPC6 plays a role primarily in proliferation and not in metastasis. However, two recent studies show that TRPC6 promotes cancer cell migration in head and neck squamous cell carcinomas (Bernaldo de Quirós et al., 2013) and glioblastoma (Chigurupati et al., 2010). In fact TRPC6 expression is increased in head and neck squamous cell carcinomas tumor samples and cancer cell lines. In this type of carcinomas knockdown of TRPC6 expression does not significantly alter cell proliferation but dramatically inhibits invasion (Bernaldo de Quirós et al., 2013). The authors showed by the means of wound-healing assays and matrigel invasion assays that the effect on invasion is much more pronounced than in migration: knock down of TRPC6 expression by siRNA resulted in a 36% decrease in cell migration and in a ~90% decrease in invasiveness. These data suggest an essential role of TRPC6 in the 3D motility of cancer cells (Bernaldo de Quirós et al., 2013). On the other hand, in glioblastoma multiforme, the most common primary brain tumor in humans and one of the most angiogenic tumors, TRPC6 expression is markedly upregulated compared to normal brain tissue. This increase in the channel expression is dependent on Notch activation under hypoxia conditions. Both pharmacological inhibition of Notch and knockdown of TRPC6 expression reduce in a similar way glioma migration and invasion *in vitro* (Chigurupati et al., 2010) (Table 1).

Beside its role on cancer cell migration, inhibition of the hypoxia-induced TRPC6 expression has an effect in endothelial cell tube formation *in vitro* as it reduced the number of branch points (Chigurupati et al., 2010), indicating that TRPC6 is essential for the angiogenic potential of glioma cells. In this regard, TRPC6 has been now largely accepted as a key player in cell migration in both macrovascular as well as microvascular EC. In particular it has been related to VEGF signaling pathway as the responsible for Ca^2+^ influx and consequent downstream effects (Cheng et al., 2006; Ge et al., 2009). Dominant negative TRPC6 significantly reduces EC number, migration and sprouting (Hamdollah Zadeh et al., 2008). Moreover, TRPC6 promotes both proliferation and tubulogenesis induced by VEGF, but not bFGF, in HUVECs (Ge et al., 2009). Phosphatase and tensin homolog (PTEN) interacts with TRPC6 and regulates cell surface expression of TRPC6 and consequently Ca^2+^ entry, endothelial permeability, and *in vitro* angiogenesis in human pulmonary arterial ECs (HPAECs) (Kini et al., 2010) (Table 1; Figure 1). Interestingly PTEN-TRPC6-mediated migration and tubule formation *in vitro* does not require the PTEN phosphatase domain, pointing out an interesting new role for PTEN as scaffolding protein (Kini et al., 2010).

Interestingly, TRPC6 can also inhibit EC migration acting in concert with TRPC5: when ECs are incubated in lysophosphatidylcholine (LPC), rapid translocation of TRPC6 initiates Ca^2+^ influx that results in externalization of TRPC5. Activation of this TRPC6–5 cascades cause a prolonged increase in intracellular Ca^2+^ concentration (Ca^2+^_*i*_) that inhibits EC movement. This effect is largely attenuated in EC harvested from aortas of TRPC6-/- mice: although LPC caused a prolonged rise in Ca^2+^_*i*_ in wild-type EC, it had no effect on Ca^2+^_*i*_ in TRPC6-deficient ECs (Chaudhuri et al., 2008) (Table 1; Figure 1). Moreover LPC-mediated TRPC6 and TRPC5 activation is mediated by tyrosine phosphorylation. This is an interesting finding since fyn and src tyrosine kinases have been described to regulates TRPC6 activity in COS-7 cells (Hisatsune et al., 2004).

Thus the final role of TRPC6 channels on EC migration is closely associated with cellular microdomains composition: when associated with VEGF receptor TRPC6 may function as downstream player and activate EC migration; on the other hand when localized in proximity with TRPC5, TRPC6-TRPC5 cascade results in attenuation of EC migration (Figure 1).

## TRPV channels

### TRPV1

A growing set of recent evidence using the agonists and antagonists of TRPV1 channel suggest that this channel could be implicated in the modulation of migration and invasion of several types of cancer cells (Waning et al., 2007; Ramer and Hinz, 2008; Ramer et al., 2010; Caprodossi et al., 2011). The most known agonist of TRPV1 capsaicin (the main component in chili pepper) has been shown to lead to an acceleration of human hepatoblastoma cells (HepG2) pretreated with hepatocyte growth factor (HGF). In contrast, HepG2 cells kept in the absence of HGF are not accelerated by capsaicin while the TRPV1 antagonist capsazepine prevents the stimulation of migration. Patch-clamp experiments of the treated cells suggest that the sustained stimulation of migration by capsaicin is probably due to a sustained elevation of TRPV1 channel activity (Waning et al., 2007) (Table 1). However, three other studies propose an anti-migratory and anti-invasive role for TRPV1 in lung, cervical and bladder cancer cell (Ramer and Hinz, 2008; Ramer et al., 2010; Caprodossi et al., 2011). In particular, in the two studies by Ramer et al., it is shown that the TRPV1 antagonist capsazepine restores invasiveness of lung and cervical cancel cell, the inhibition of which is due to cannabinoid treatment. It has to be noted that cannabinoids were shown to have an antitumorigenic role inhibiting cell metastasis and angiogenesis (Portella et al., 2003; Blázquez et al., 2004) most likely due to stimulation and consequent desensitization of TRPV1 channel (De Petrocellis et al., 2011). The molecular mechanism of this anti-invasive effect was further studied and it was shown that the TRPV1 antagonist capsazepine causes a significant suppression of both cannabidiol-induced tissue inhibitors of the matrix metalloproteinase 1 (TIMP-1) expression and activation of the MAPKs p38 and p42/44 (Ramer et al., 2010) (Figure 1).

TRPV1-mediated inhibition of migration has been analyzed more in detail in a recent study in bladder cancer (Caprodossi et al., 2011) (Table 1; Figure 1). In this study two urothelial cell lines were used, the low grade RT4 cells and the moderately differentiated 5637 cells of a higher grade in which TRPV1 mRNA and protein levels are dramatically reduced as compared with RT4 cells. Capsaicin promotes the invasiveness of 5637 cells by triggering IGF (insulin-like growth factor)-1 release, GZMA and MMP9 activation, a-tubulin disassembly and cytoskeleton degradation. Interestingly, TRPV1 transfection of these cells reverts the capsaicin-induced migration and MMP9 activation, suggesting an inhibitory role played by TRPV1 in urothelial cancer cell invasion and metastasis.

### TRPV2

TRPV2 is expressed in aggressive prostate and bladder cancer cells and tissue samples in which its activation stimulates the migration and invasive phenotype of these cells (Caprodossi et al., 2008; Monet et al., 2009, 2010). In particular two factors, lysophospholipids and adrenomedullin, were shown to increase cell motility by activating the channel. Lysophospholipids are significant actors in tumor development, since they stimulate angiogenesis, growth, survival and migration of malignant cells from various origins (Raj et al., 2004; Hao et al., 2007). Likewise lysophospholipids (LPC and lysophosphatidylinositol, LPI) were shown to act as new physiological stimuli for TRPV2 channel inducing channel translocation to the plasma membrane through activation of Gi or Go proteins and phosphatidylinositol 3,4-kinase (PI3,4K signaling). This accumulation of the TRPV2 channel in the plasma membrane results into higher Ca^2+^ entry which in turn promoted prostate cancer cell migration by induction of key invasion markers, such as the matrix metalloproteinases MMP2, MMP9 and cathepsin B52 (Monet et al., 2009). Further it was shown that siRNA- mediated TRPV2 silencing reduces the size and invasive properties of xenografted prostate tumors in nude mice and downregulates the expression of MMP2, MMP9 and cathepsin B52, suggesting that TRPV2 is a viable pro-metastatic target *in vivo* (Monet et al., 2010) (Table 1; Figure 1).

The second factor characterized so far acting on cell migration through TRPV2 is adrenomedullin, a peptide originally isolated from a human pheochromocytoma (Kitamura et al., 1993). A number of studies have implicated adrenomedullin in tumor growth, progression and metastasis by affecting angiogenesis, cell proliferation, apoptosis and migration (Zudaire et al., 2003; Nakamura et al., 2006; Nikitenko et al., 2006). In this regard, it was recently shown that adrenomedullin, induces prostate and urothelial cancer cell migration and invasion through TRPV2 translocation to plasma membrane and the subsequent increase in resting Ca^2+^ level (Oulidi et al., 2013) (Table 1; Figure 1).

### TRPV4

TRPV4 is an interesting emerging player in cell migration. In particular, an increasing amount of literature is accumulating on vascular EC: the high selectivity of the pharmacological compounds acting as antagonists for this channel makes it a promising molecular target for antiangiogenic treatments (Everaerts et al., 2010). TRPV4 is widely expressed in the vascular endothelium where it is proposed to act as a mechanosensor. The channel is indeed activated by changes in cell morphology, during cell swelling and shear stress (Vriens et al., 2004; Everaerts et al., 2010). In particular fluid shear stress regulates cell re-orientation in a TRPV4 dependent manner, while in larger arteries the channel is a key player in shear stress-induced vasodilation (Hartmannsgruber et al., 2007; Thodeti et al., 2009). More recently the molecular mechanism underlie ultrafast TRPV4 by shear stress has been investigated: mechanical force applied through b1 integrin induces a near instantaneous and localized transient TRPV4 mediated Ca^2+^ influx in intact capillary EC expressing both native and genetically engineered integrin receptors. The ultra rapid response of the Ca^2+^ signal (within 4 msec) observed using whole cell Ca^2+^ imaging strongly suggests that TRPV4 channels are activated in the absence of second messengers, and are directly mechanosensitive (Matthews et al., 2010). Both shear stress and agonist-activation of TRPV4 enhance EC proliferation and trigger collateral growth after arterial occlusion (Troidl et al., 2009). Fiorio Pla et al. recently demonstrated that AA-activated TRPV4 is essential for breast tumor-derived EC (BTEC) migration: the expression of endogenous TRPV4 was significantly higher in BTEC compared to “normal” EC (HMEC) (Fiorio Pla et al., 2012b). TRPV4 plays a key role in mediating Ca^2+^ entry in BTEC as loss of TRPV4 expression resulted in complete inhibitions of Ca^2+^_*i*_ responses and migration induced by the specific agonist phorbol ester 4α-phorbol 12,13-didecanoate (4αPDD) and AA. Finally, AA induces actin remodeling and increases surface expression of TRPV4 in BTEC (Fiorio Pla et al., 2012b) (Table 1; Figure 1). It is important to stress the use of BTECs in this study as suitable model to investigate the role of proangiogenic factors and their related cell signaling triggered in ECs, compared to the widely used ECs obtained from normal tissues. In fact the great amount of detailed studies on proangiogenic endothelial signaling have been performed *in vitro* on different types of primary or immortalized macrovascular and microvascular EC lines from human or animal normal tissues; on the other hand much less information is available so far about tumor-derived ECs (Fiorio Pla et al., 2012a). As previously reported for TRPC6, the dynamics of a single TRP should be considered in a more integrated framework: in this regard recent data reported that the trafficking to the plasma membrane of TRPV4-TRPC1 heteromeric complex is enhanced by Ca^2+^ store depletion in HUVEC, resulting in an enhanced Ca^2+^ influx upon exposure to shear flow (Ma et al., 2010). Moreover, enhanced TRPV4-C1 trafficking to the plasma membrane contribute to SOCE and I_SOC_ in the EC (Ma et al., 2011). On the other hand, after a heated debate, it is now largely accepted thet TRPC1 channels contribute to SOCE in different cells type including HUVECs (Cheng et al., 2013).

As regarding cancer cells, not much is known about its role in migration. However a study from Schwab and co-workers has described a role for TRPV4 in hepatoblastoma cell line (HepG2): 4αPDD led predominantly to increased lamellipodial dynamics and velocity in HGF treated HepG2 cells, although the displacement, a measure of the cell persistence, was not statistically different from control conditions (Waning et al., 2007).

## TRPM channels

### TRPM1

TRPM1 has been suggested to be a tumor suppressor and a decrease in TRPM1 expression appears to be a prognostic marker for metastasis in patients with localized malignant melanoma (Duncan et al., 1998; Fang and Setaluri, 2000). Taking in consideration the loss of expression of TRPM1 channel during the progression of melanomas toward more invasive forms it could be hypothesized that TRPM1 presence and/or activation inhibits migration. A very interesting recent study shed light to that issue and the authors report that tumor suppressive activity is not mediated by TRPM1 directly but by a microRNA (miR-211) hosted within an intron of TRPM1. Increasing expression of miR-211 but not TRPM1 reduces migration and invasion of malignant and highly invasive human melanomas characterized by low levels of melastatin and miR-211. Thus this intronic miRNA assumes a tumor suppressive function previously ascribed to *trpm1*, its host gene (Levy et al., 2010). However, miRNA genes are frequently hosted by protein coding genes, phenotypes attributed to genetic deletion of protein-coding genes may actually be attributable to abrogated expression of the hosted miRNAs (Moffett and Novina, 2007). It has to be noted that a single miRNA may target thousands of genes: it is therefore possible that altered expression of a single miRNA can regulate complex phenotypes. Indeed, a network analysis of melanoma-expressed genes filtered for their roles in metastasis identified three central node genes: IGF2R, TGFBR2, and NFAT5 (Levy et al., 2010). Expression of these genes is reduced by miR-211 and knockdown of each gene phenocopied the effects of increased miR-211 on melanoma invasiveness. In conclusion, this study suggest miR-211 as a suppressor of melanoma invasion whose expression is silenced (or selected against) via suppression of the entire melastatin locus during human melanoma progression (Levy et al., 2010).

### TRPM7

TRPM7, as TRPV4, is another stretch-activated Ca^2+^ and Mg^2+^ permeable channel, which present a kinase domain at its C-terminus and allowing its involvement both in cellular Mg^2+^ status and in intracellular signaling (Bates-Withers et al., 2011; Paravicini et al., 2012). TRPM7 has been described as a regulator of actomyosin contractility, cell adhesion, and directed cell migration (Clark et al., 2006, 2008; Su et al., 2006; Prevarskaya et al., 2011; Schwab et al., 2012). In particular TRPM7 is responsible for the Ca^2+^ flickers at the front lamellipodia of migrating fibroblast mediating the polarized migration toward a chemo attractant (Wei et al., 2009). However, a role for this bifunctional channel in cancer progression has been proposed only recently: TRPM7 is critical for cell migration of different cell cancer models such as breast cancer, lung cancer, nasopharingeal cancer and pancreatic ductal adrenocarcinoma (Chen et al., 2010; Gao et al., 2011; Middelbeek et al., 2012; Rybarczyk et al., 2012; Meng et al., 2013). Silencing TRPM7 with specific small interfering RNA lead to a significant reduction in migration and invasion capability of MDA-MB-435 via Src and p38, JNK and ERK1/2 signaling pathway (Meng et al., 2013) as well as by regulating myosin II–based cellular tension, thereby modifying focal adhesion number, cell–cell adhesion and polarized cell movement (Middelbeek et al., 2012) (Table 1; Figure 1). Interestingly in lung cancer cells A549, EGF functionally regulates TRPM7 expression at the plasma membrane thus increasing cell migration rate (Gao et al., 2011) while TRPM7 protein downregulation significantly interferes with the metastatic potential of human breast cancer *in vivo* (Middelbeek et al., 2012).

On the other hand TRPM7 is involved in a number of vascular disorders such as hypertension and dysfunction of endothelial and smooth muscle cells (Yogi et al., 2011).

Opposite data have been reported for different ECs: while TRPM7 silencing significantly impairs HMEC motility (Baldoli and Maier, 2012), TRPM7 inhibition stimulates HUVEC to migrate (Baldoli et al., 2013). These results further underscores that TRPM7 serves different functions in EC of different origins. On possible explanation for this descrepancy is that HUVEC and microvascular EC might express different Mg transporters. In particular, Baldoli and coworkers discuss the possibility that other Mg channels vicariate TRPM7 functions in HUVEC.

### TRPM8

TRPM8 expression is strongly up-regulated in numerous cancers such as prostate, breast, colon, lung, pancreas and skin while it is dramatically reduced during metastasis in the androgen-independent prostate cancer (Tsavaler et al., 2001; Henshall et al., 2003; Yee et al., 2010). This pattern of variation in TRPM8 channel expression makes it an interesting candidate as a diagnostic marker for detection of cancer and as prognosis marker for evaluating the outcome of epithelial cancers (Zhang and Barritt, 2006). Concerning the role of this channel in migration Gkika et al have recently propose that it could have a protective role in prostate metastatic cancer (Gkika and Prevarskaya, 2011), since recent data show that it blocks cancer cell migration in prostate cells (Yang et al., 2009b; Gkika et al., 2010; Zhu et al., 2011; Wang et al., 2012). In particular overexpression of TRPM8 in prostate cancer cells reduces the cell motility through the inactivation of FAK (Yang et al., 2009b) (Figure 1). Moreover, it seems that only the presence of TRPM8 on the plasma membrane is sufficient to reduce migration, suggesting a basal activity of the channel possibly affecting FAK phosphorylation while TRPM8 activation by icilin, one of its agonists, further reduced cell motility (Gkika et al., 2010). Interestingly, the well-known prostate cancer marker, Prostate Specific Antigen (PSA) that is secreted in the prostatic acini and is therefore in contact with the extracellular part of TRPM8, activates the channel and decelerates cell migration by inducing its plasma membrane accumulation (Gkika et al., 2010) (Table 1; Figure 1). Furthermore, TRPM8 expression has a negative effect in angiogenesis as it was recently shown in nude mice. Mice transplanted with prostate cancer cells over-expressing TRPM8 develop tumours that are less vascularized than control. The lower microvascular density of the TRPM8 xenografts can be explained by their lower expression of FAK and VEGF, which is one of the most potent angiogenic factor (Zhu et al., 2011). Taken together these three studies suggest that TRPM8 could play a protective role in prostate cancer progression by reducing both cell migration and angiogenesis.

It has to be noted that contrasting results on the role of TRPM8 in cell migration have been shown in glioblastoma, prostate cancer and squamous carcinoma cell lines (Wondergem et al., 2008; Okamoto et al., 2012; Valero et al., 2012;). In particular, Nilius and co-workers suggest that menthol (TRPM8 agonist) accelerates cell migration of glioblastoma cells (Wondergem et al., 2008); on the other hand, pharmacological agents inhibiting TRPM8 reduce cell speed of prostate cancer cells (Valero et al., 2012). However, the authors used a pharmacological approach and no direct data using siRNA or overexpression that would reinforce the involvement of the channel in cell migration was provided. The discrepancy in the results in between these different studies can be due to several reasons: (i) the pharmacological agents used are not the same, (ii) agonists/antagonists can involve the activation of other channels (or proteins), (iii) the cell systems were not always the same.

It would be interesting to investigate the *in vivo* effects of the activators and inhibitors of TRPM8, as well as the use of pore mutants in addition to the wild-type channel overexpression used in mice xenografts (Zhu et al., 2011). These experiments together with *in vitro* studies could give some insight on which is the critical factor in migration during carcinogenesis: the activity of the TRPM8 channel or the changes in conformation of the TRPM8 protein during its interaction with pharmacological agents and its subsequent changes in protein-protein complexes? However, the present divergence in the results is as puzzling as in the case of TRPV1 and makes it difficult to draw conclusions concerning the use of the channel agonists or antagonists as pharmacological candidates in clinics.

## ORAI1/STIM1 complex

Orai1 and STIM1 are components of the so-called Ca^2+^ release activated currents (CRAC) channels (Yeromin et al., 2006; Cahalan et al., 2007; Hewavitharana et al., 2007). Since they are closely linked to TRP channel function, we will include the discussion for these proteins here. In this regard Recently, TRPC proteins have been shown to associate with Orai1 and STIM1 in a dynamic ternary complex regulated by the occupation of membrane receptors in several cell models, which might play an important role in the function of TRPC proteins (Salido et al., 2011; Cheng et al., 2013).

Reflecting the eminent importance of CRAC current following receptor stimulation, several recent studies addressed the role of Orai1/STIM1 in chemotactically or chemokinetically stimulated migration with a particular focus on cell adhesion both in cancer cells as well as in vascular endothelium. Orai1/STIM1 complex is implicated in breast, nasopharyngeal carcinoma, cervical and glioblastoma multiforme tumor cell migration *in vitro* and in a mouse model of metastases generated by tumor xenografts (Yang et al., 2009a; Chen et al., 2011; Motiani et al., 2013; Zhang et al., 2013). The inhibition of store-operated Ca^2+^ entry (SOCE) by a pharmacological agent, SKF96365, or by siRNA-mediated STIM1 or Orai1 silencing is able to inhibit MDA-MB- 231 cell migration and matrigel invasion, as well as reduce the spread of xenografted tumor cells in mice; on the other hand, reexpression of siRNA-resistant STIM1 or Orai1 constructs rescued the invasion of the STIM1 or Orai1 siRNA-treated cells. STIM1- and Orai1-mediated SOCE regulates cell migration at least partly through modulating focal adhesion turnover, which in turn facilitates cell migration of metastatic cancer cells; on the other hand blocking Ca^2+^ influx affects both the assembly and disassembly of focal adhesions, which may impair traction force generation in migrating cells. The defects of focal adhesion turnover and cell migration induced by SOCE impairment can be rescued by constitutively active forms of the small GTPases RAS and RAC, suggesting the involvement of these regulators of focal adhesions in the modulation of cell migration by SOCE (Yang et al., 2009a) (Table 1; Figure 1). Similar results were obtained on hepatoblastoma cell line where STIM1 play a key role in focal adhesion turnover (Yang et al., 2013). STIM1 is also a key player in EGF-mediated cervical cancer and nasopharyngeal carcinoma cell migration by inhibiting calpain activity and focal adhesion turnover (Chen et al., 2011; Zhang et al., 2013); STIM1 play also a role in stimulating angiogenesis by regulating VEGF-A release from cancer cells thus proposing a multiple function for STIM1 in tumor biology (Chen et al., 2011). On the other hand recently the association of Orai1 and K+ channels have been involved in cancer cell migration: SK3 channels functionally associate with the Orai1 channel in a breast cancer MDA-MB-435s within lipid rafts. This localization of an SK3–Orai1 complex seemed essential to control cancer cell migration and bone methastases. Interestingly, STIM1 seems not to be involved in this effect (Chantôme et al., 2013) (Figure 1).

The role of ORAI1/STIM1 complex has been studied recently also in EC migration and tumor vascularization. In particular it concurs to the VEGF-mediated SOCE in HUVECs (Abdullaev et al., 2008; Li et al., 2011). VEGF stimulation promotes STIM1 clustering which in turn activates Orai1 (Li et al., 2011). Moreover, knock-down of Orai1 inhibits HUVEC migration, proliferation and *in vitro* tubulogenesis subsequent to the sustained intracellular Ca^2+^ elevation triggered by VEGF (Abdullaev et al., 2008; Li et al., 2011; Beech, 2012) (Figure 1). Interestingly, it has been recently reported that STIM1, as well as TRPC1 and TRPC4 knockdown, reduces tube formation in both HUVECs and EA.hy926 cells (Antigny et al., 2012). These data are of particular interest since functional interaction between Orai1 and TRPC1 has been described as previously stated (Ong et al., 2007; Salido et al., 2011; Cheng et al., 2013).

Notably, suppression of Orai1 expression or expression of dominant negative mutants of Orai1 abolish SOCE in EPCs as well as *in vitro* tubule formation (Li et al., 2011). Blockade of SOCE affects EPC proliferation, migration and *in vitro* tubulogenesis induced by VEGF (Dragoni et al., 2011). Moreover, EPCs isolated from RCC patients (RCC-EPCs) display a greater SOCE, which correlates with the over-expression of Orai1, Stim1, and TRPC1 as compared to control cells (EPCs from healthy patient) (Lodola et al., 2012).

## Conclusions

In the two last decades the progressive understanding of the molecular mechanisms that regulate the establishment and progression of different tumors is leading to even more specific and efficacious pharmacological approaches. In this picture, TRP channels represent a very promising player since their expression and activity mark and regulate specific stages of cancer progression (Gkika and Prevarskaya, 2009; Prevarskaya et al., 2011; Schwab et al., 2012; Ouadid-Ahidouch et al., 2013). On the other hand, the downside of migration is represented by disease conditions in which cell migration constitutes a major pathophysiological element. The most prominent example is the formation of tumor metastases. Tumor cell migration is an essential step of the so-called metastatic cascade that leads to the spread of the disease within the body (Yamaguchi et al., 2005; Lohela and Werb, 2010).

The data presented in this review firmly proof the concept that different members of the TRP family of channels constitute integral components of the cellular migration machinery linking in many cases the plasma membrane (or intracellular membranes) signals to cytoskeletal migration motor or cell adhesion proteins functions in cancer cells. Indeed, locomotion involves lamellipodial extensions at the leading edge, new attachments to stabilize the extension, transcellular contractility, and detachment at the rear. These processes involve a multitude of molecules such as growth factors and their receptors, matrix metalloproteinases, integrin, small GTPases, FAK and SRC kinases, elements of the cytoskeleton, all of which underlie biochemical events of the moving cells. In particular, data on TRPM7 and Orai1/STIM1 complex clearly link these channels to this dynamic process leading to directional migration (Yang et al., 2009a; Middelbeek et al., 2012). It is also clear that the study of TRP channels in cellular migration is a young field (as denoted by the fact that the majority of data are from the last 10 years) so that studies mechanistically linking the channel role to the migration machinery are still limited and more research is needed. On the other hand, the scientific interest on this topic is largely increasing as pointed out by PubMed search and we aspect the knowledge of the molecular mechanisms responsible for TRP channels role in migration will be largely unveiled in the next ten years.

Another issue to be considered is the wide distribution as well as the multiplicity of cancer hallmarks controlled by a given TRP or Orai1 protein which requires careful consideration of its therapeutic potential. This problem could be overcome by directed targeted therapies taking advantage from nano-biomedicine: for example, nanoparticle functionalization with peptide cyclic RGD for angiogenesis-specific targeting (Cheng et al., 2011) together with a specific channel modulator could be successfully employed. Another good example of such a strategy is the ‘smart bomb’ for prostate cancer, which combines a sarcolemmal and endoplasmic reticulum Ca^2+^-ATPase (SERCA) inhibitor thapsigargin (which induces apoptosis through the activation of ER stress and Ca^2+^ entry pathways) with a targeting peptide that is a substrate of the serine/protease prostate-specific antigen (PSA) (Denmeade and Isaacs, 2005).

The wide expression of TRP channels has also to be taken in account regarding vascular endothelium. In this regard when considering TRPV4 potential role in tumor vascularization (Fiorio Pla et al., 2012b) it’s important to notice that TRPV4 is ubiquitary in healthy vascular endothelium and plays a physiological role both in large arteries and microvessels: these relevant activities require careful consideration of its therapeutic potential. On the other hand, an overexpression on TEC could be exploited for a tumor targeted therapy based on lower inhibiting doses of TRPV4 antagonists which could selectively affect TEC and not normal EC.

Moreover ubiquitous expression of the channels could be used as a positive feature, due to the redundancy of the signaling pathways which regulates the different hallmarks of cancer: in other words, the use of specific channels to selective co-target different key steps of carcinogenesis beside tumor vascularization, could result in more effective and long lasting therapies. For example, TRPC6 channels targeting could affect VEGF release from tumor cells as well as EC migration and tumor vascularization (Hamdollah Zadeh et al., 2008; Ge et al., 2009; Chigurupati et al., 2010).

In addition as it was described above for some channels (TRPV1 and TRPM8) pharmacological action has not always the same result with siRNA and/or overexpression approaches due to broad action on several cellular components. There is therefore a real need for detailed studies *in vivo* in order to test which are the most effective and suitable molecules to target in therapeutics. Another reason for the possible differences in the cellular responses observed in different tumors could be due to some peculiar features of these channels in certain cellular environments. It is now well described that TRP channel can interact in specific microdomains giving rise to different signal transduction pathways and cellular signals. In this regard an example is given by TRPC6: when associated with VEGF receptor TRPC6 may function as downstream player and activate EC migration (Chigurupati et al., 2010); on the other hand when localized in proximity with TRPC5, TRPC6-TRPC5 cascade results in attenuation of EC migration (Chaudhuri et al., 2008). On the other hand TRP and ORAI1 channels functionally interact with other family of channels: a nice example is TRPC1 which is functionally related to ClC-3 in caveolar lipid rafts of glioma cells to promote EGF-induced chemotaxis (Cuddapah et al., 2013). Similarly ORAI1 interacts with K+ channels in breast cancer (Chantôme et al., 2013). Differences in channels complexes and/or microdomain localization could therefore account for different cellular effect in tumors.

In conclusion we expect that the more detailed understanding together with a more integrative view on TRP channels in cell migration, as well as careful studies using appropriate *in vivo* and *in vitro* models, will facilitate the advances in this exiting field of cellular physiopathology

### Conflict of interest statement

The authors declare that the research was conducted in the absence of any commercial or financial relationships that could be construed as a potential conflict of interest.
